# Development of artificial intelligence and multi-sensor-based dexterity assessment system: performance evaluation

**DOI:** 10.1007/s11517-025-03382-2

**Published:** 2025-06-16

**Authors:** Mehmet Emin Aktan, Sena Zeybek Kılıç, Erhan Akdoğan, Tuğçe Özekli Mısırlıoğlu, Deniz Palamar

**Affiliations:** 1 Department of Mechatronics Engineering, Bartın University, 74110 Bartın, Türkiye; 2 Health Institutes of Türkiye, 34718 İstanbul, Türkiye; 3 Department of Mechatronics Engineering, Yıldız Technical University, 34349 İstanbul, Türkiye; 4 Department of Physical Medicine and Rehabilitation, Istanbul University-Cerrahpasa, 34098 İstanbul, Türkiye

**Keywords:** Wearable sensors, Dexterity test, Electromyography, Artificial neural networks, Image processing

## Abstract

Manual dexterity tests are essential for diagnosing diseases and evaluating professional skills that require fine motor control. Traditional assessments often depend on expert supervision, leading to delays, subjectivity, and inaccuracies. This study introduces an automated dexterity assessment system that integrates multiple sensors and artificial intelligence algorithms for high-precision evaluation. The system classifies hand movements, analyzes muscle contraction levels, and determines hold-release durations using electromyography (EMG), inertial measurement units (IMU), and image processing techniques. An expert system interprets the multimodal sensor data and presents the results to clinicians. A performance evaluation with 20 participants demonstrated the system’s capability to assess hand dexterity automatically and accurately.

**Graphical Abstract:**

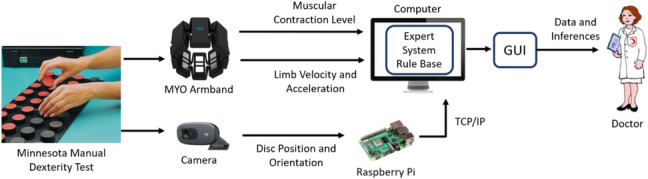

## Introduction

The hand is one of the most used parts of the upper limb in daily life activities. Manual dexterity, defined as the ability to perform precise and coordinated movements with the hands and fingers, is crucial for performing various daily and occupational tasks. Loss of dexterity can result from conditions such as nerve injury, amputation, arthritis, fractures, carpal tunnel syndrome, muscular dystrophies, and neurological disorders [1]. The physical dimensions of the hand and fingers also affect dexterity [2, 3]. In addition, studies have shown that dexterity declines with aging [4–7]. Evaluation of manual dexterity is important in determining the functionalities and limitations in daily life activities. In terms of physiotherapy, it is necessary to determine the loss of function in the hand to determine and apply the appropriate treatment. Several standardized dexterity tests have been developed for these purposes, including the Jebsen–Taylor Hand Function Test [8], the Purdue Pegboard Test [9], the Grooved Pegboard Test, the Crawford Small Parts Dexterity Test [10], and the Minnesota Manual Dexterity Test (MMDT). These tests are widely used not only for medical assessment but also for personnel selection in industries requiring fine motor skills.

However, conventional dexterity tests have several limitations. First, they require supervision by an expert, which can lead to scheduling delays and inefficiencies in healthcare settings. Second, these tests rely primarily on total task completion time as the evaluation criterion, offering limited insights into the subcomponents of manual dexterity, such as cognitive processing time, grasping ability, and manipulation skills. Third, human supervision introduces potential measurement errors and subjectivity into the assessment process. Furthermore, individuals being tested may experience anxiety in the presence of an evaluator, which can negatively affect their performance.

### Limitations of existing methods and research gaps

In recent years, researchers have attempted to develop electronic and sensor-based systems to enhance dexterity assessment. Several approaches have been explored:

#### **Electronic pegboard tests** 

Acharya et al. [11] developed an electronic Nine-Hole Peg Test that detects peg insertion using light-dependent resistors (LDRs) and LEDs. However, this system only records completion time and does not analyze movement patterns or hold-release behavior. Al-Naami et al. [12] designed an electronic Grooved Pegboard Test with switches in the peg holes to measure placement time. This setup lacks motion profiling and fails to assess complex hand–eye coordination skills due to its simplified circular hole design. Okahashi et al. [13] introduced a system that tracks peg placement using magnetic switches but does not capture detailed movement kinematics or muscle activity.

#### **Wearable sensor-based systems** 

Chuang et al. [14] and Rashid and Osman [15] developed dexterity assessment systems using sensor-integrated gloves. While these systems provide additional motion data, the use of gloves restricts hand movement, reducing natural dexterity. Hsiao et al. [16] developed a glove with an IMU sensor and force-sensitive resistors for dexterity assessment. Although flexible sensors are useful for determining joint range of motion, they have the disadvantages of wearing out in a short time, requiring calibration and distorting the nature of the movement. The biggest disadvantage of glove-based solutions is that gloves cause movement restriction. Riddle et al. [17] evaluated the finger forces of individuals during daily activities with pressure sensors placed on the fingers. They also investigated the effect of these sensors on the completion time of the tasks. It was found that subjects completed tasks with large objects 13% slower, medium-sized objects 31% slower, and small objects 41% slower when sensors were attached to the fingers. Woods [18] investigated the effect of wearing gloves on skill performance while undergoing MMDT. It was observed that gloves increased the test time by 35%. These results show that materials such as sensors, apparatus, cables, and gloves worn on the hand have a negative effect on dexterity.

Recent deep learning-based methods for multivariate time series classification, such as densely knowledge-aware network (DKN) [19], deep transformer capsule mutual distillation (DTCM) [20], CapMatch: semi-supervised contrastive transformer capsule [21], and deep contrastive representation learning with self-distillation [22], have shown significant performance in activity recognition and classification tasks. However, these methods primarily focus on learning global representations from time series data rather than providing explicit inference about dexterity subcomponents. While these approaches leverage feature-based knowledge distillation and self-supervised learning to improve classification accuracy, they do not incorporate multimodal sensor fusion in a way that supports detailed movement profiling for dexterity assessment. In contrast, our proposed system integrates electromyography (EMG), inertial measurement unit (IMU), and computer vision-based motion tracking to extract granular insights into hand dexterity beyond classification tasks.

### Improvements and contributions of the proposed system

To address these limitations, this study presents a multi-sensor-based dexterity assessment system that integrates electromyography (EMG), inertial measurement unit (IMU), and computer vision-based motion tracking. The key contributions of this work are:

- **Automated multi-sensor integration:** Unlike previous works that rely on a single measurement approach, our system integrates EMG, IMU, and computer vision to capture muscle activity, movement kinematics, and dexterity subcomponent durations.
- **Detailed movement and cognitive analysis:** The system not only determines total test duration but also determines subtask durations (grasping, manipulation, and release) to help identify which aspect of dexterity is impaired.
- **Expert rule-based system for decision support:** The extracted sensor data is evaluated through an expert system that interprets dexterity impairments and provides clinically relevant insights, reducing the need for manual evaluations.
- **Validation with healthy participants:** The system’s performance was tested with 20 participants, demonstrating high accuracy in motion classification (96.2%) and dexterity stage recognition, ensuring reliable clinical applicability.

By integrating these advances, the proposed system provides a comprehensive, objective, and automated dexterity assessment method, addressing the limitations of traditional and existing electronic systems. The developed framework enables more accurate diagnoses, personalized rehabilitation plans, and enhanced efficiency in clinical and occupational assessments.

## Material and method

**Fig. 1:**
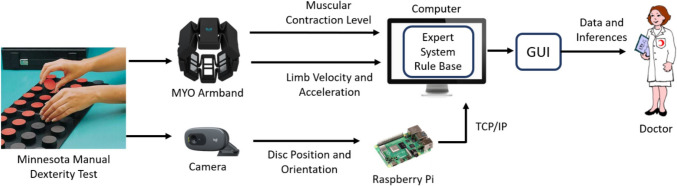
General block diagram of the system

The general block diagram of the system is given in Fig. 1. The system structure, the general concept of which has been revealed [23], was realized. The study was approved by the Istanbul University-Cerrahpaşa Clinical Research Ethics Committee (E-83045809–604.01.01–374132). During the MMDT, muscle contraction levels and limb velocities and accelerations are obtained through the MYO armband placed on the forearm. These are processed in MATLAB on a computer (Intel(R) Core(TM) i9-12950HX CPU @ 2.30 GHz 64 GB RAM) to obtain the times of the hold-release movement and the angular velocity and linear acceleration of the limb. The user’s placement time of the discs is determined by image processing. This process was done with Python software running on Raspberry Pi 4. In addition, the performance of the users is evaluated with the rule base, which is created with the information received from the experts, and inferences regarding the physical and cognitive status of the participant are made. In experiments, the time taken for all processes is about 140–290 ms. The details of these measurement processes, the system integration, and the detailed working principle are explained in this section.

### Determination of disc placing and turning times

In the study, the MMDT was used as a dexterity test setup. This test method is widely used to measure the performance of healthy hands and to monitor the patient’s progress in rehabilitation [24, 25]. The test includes 60 discs and a test board on which these discs will be placed. One surface of the discs is black, and the other surface is red. In the test, the discs must be placed in a certain order and sequence. There are two phases in the test: placing and turning. The participant is informed that time is important.

**Fig. 2:**
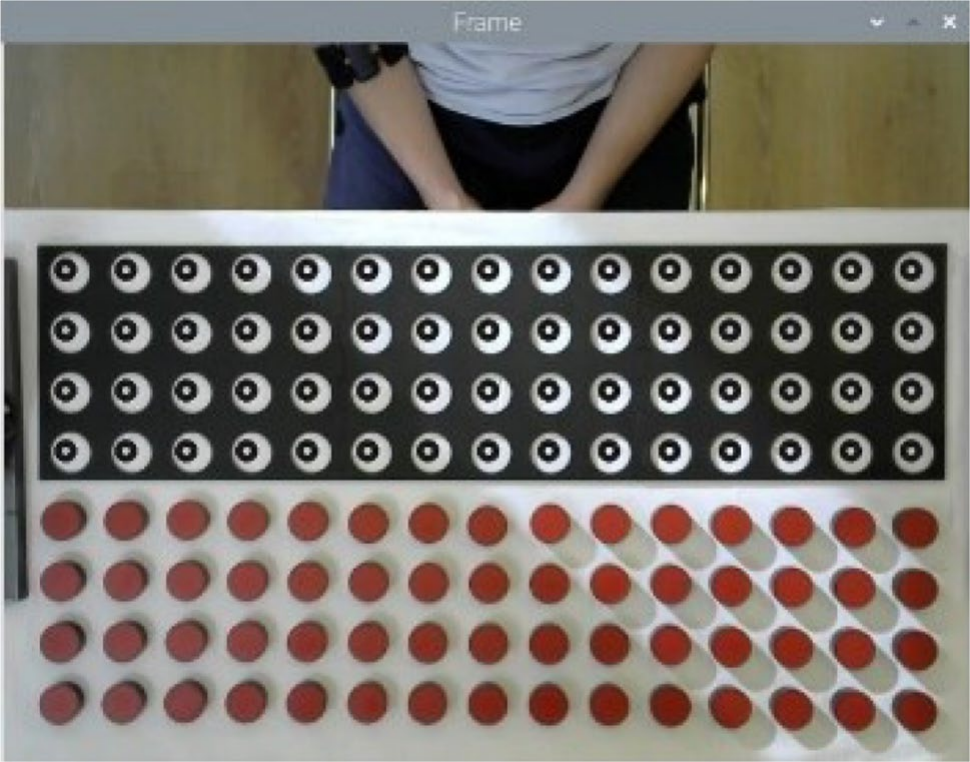
Marking hole centers on the test board

During the MMDT, image processing was used to determine the placement of the discs on the test table and the placement and rotation times of each disc. The images obtained with the Logitech C505 camera were processed in real time using the OpenCV library in Python. Images have a resolution of 640 × 480 pixels and are recorded in RGB color space. The locations of the holes on the test board where the discs were placed were marked with circles (Fig. 2). In this way, pixels with certain coordinates are scanned instead of all pixels in the camera’s field of view. Inferences were made based on the color of the pixels in the center of these holes. The table floor is white, and the disc surfaces are black and red. When the test starts, cv2.VideoCapture() is used to capture video images. cv2.COLOR_BGR2HSV is used to convert BGR to HSV. If the color of the target pixels is white, it means that there is no disc in the target yet, if the target pixel is red during the placing phase, it means that the disc is inserted correctly, and if the pixel value is in the black color range during the placing phase, it means that the disc is inserted upside down. In the turning phase, if the target pixel is red, the disc has not yet been turned; if the target pixel is black, the disc has been turned correctly; if the target pixel is white, the disc has been removed incorrectly. The color detection process was mathematically formulated using the HSV color space. Given an RGB image, the transformation to HSV is represented as

1 $$H=\left\{\begin{array}{ll}60^0\frac{G-B}{\max\;\left(R,G,B\right)-\min\;(R,G,B)}&if\;\max\;\left(R,G,B\right)=R\\60^0(2+\frac{G-B}{\max\;\left(R,G,B\right)-\min\;\left(R,G,B\right)})&if\;\max\;\left(R,G,B\right)=G\\60^0(4+\frac{G-B}{\max\;\left(R,G,B\right)-\min\;\left(R,G,B\right)})&if\;\max\;\left(R,G,B\right)=B\end{array}\right.$$

where $H$ is the hue angle in degrees. The thresholding for detecting red and black discs was determined by defining pixel intensity ranges in the HSV color space:

2 $$\begin{array}{l}T_{white}=\left\{H\in\left[0,180\right],S\in\left[0,30\right],V\in\lbrack220,255\rbrack\right.\\T_{red}=\left\{H\in\left[0,10\right]\cup\left[160,180\right],S\in\left[100,255\right],V\in\lbrack30,255\rbrack\right.\\T_{black}=\left\{H\in\left[0,180\right],S\in\left[0,255\right],V\in\lbrack0,30\rbrack\right.\end{array}$$

### Analysis of EMG signals and motion classification

**Fig. 3:**
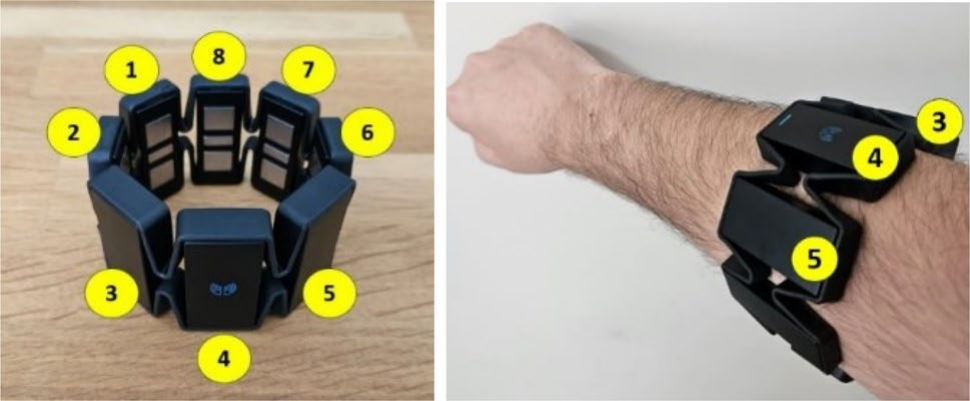
MYO armband and channel numbers

In the system, the holding and releasing times of the discs were recorded, and it was determined how long the participant held the discs during the test. For this purpose, EMG signals from the forearm were evaluated and classified. The amplitude range of an EMG signal is 0–10 mV before amplification. The EMG signal has the most frequency power between 20 and 250 Hz. The artificial neural network-based LLGMN (log-linearized Gaussian mixture model) algorithm developed by Tsuji et al. [26] was used for motion classification. EMG signals were obtained via the Bluetooth communication protocol with the MYO armband (Fig. 3).

With the 8-channel electrodes on the MYO armband, which can be worn on arms of different sizes with its easy-to-wear and elastic structure, muscle contraction signals are received and pre-processed. These processes are amplification, rectification, and filtering, respectively. Since the amplitude of raw EMG signals is very low, 20 dB amplification is performed. Then the negative alternans are converted to positive alternans by rectification. Finally, a 2nd-order low-pass Butterworth filter with a corner frequency of 3 Hz was used to remove noise from the signal.

**Fig. 4:**
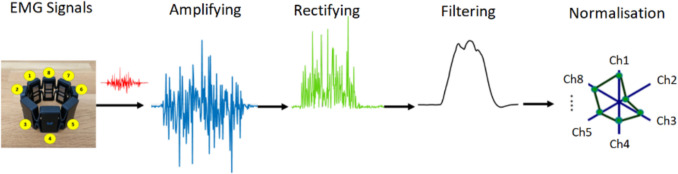
EMG signal processing stages

The sampling frequency of the MYO Armband device is 200 Hz. Sampled signals are defined as ${\text{EMG}}_{i}\left(t\right) (i=8)$. ${\text{EMG}}_{\text{i}}\left(t\right)$ signal is normalized so that the sum of the signals from the 8 channel electrodes is 1. The normalized EMG signal (${{EMG}^\prime}_i(t$)) is defined in Eq. 3.

3 $${\text{EMG}}_{i}^{\prime}\left(t\right)=\frac{{\text{EMG}}_{i}\left(t\right)-{\text{EMG}}_{i}^{\text{rest}}}{\sum_{i=1}^{L}({\text{EMG}}_{i}\left(t\right)-{\text{EMG}}_{i}^{\text{res}t})} \left(L=8\right)$$

where ${\text{EMG}}_{i}^{\text{rest}}$ is the mean of ${\text{EMG}}_{i}\left(t\right)$ in the uncontracted, released state of the arm. A diagram of EMG signal processing is given in Fig. 4.

In order to perform motion classification, the muscular contraction level (MCL) needs to be calculated using the processed EMG signals. The MCL is calculated using Eq. 4.

4 $$\text{MCL}\left(t\right)=\frac{1}{N}\sum\nolimits_{n=1}^{N}\frac{{\text{EMG}}_{n}\left(t\right)-{\text{EMG}}_{n}^{\text{rest}}}{{\text{EMG}}_{n}^{\text{max}}-{\text{EMG}}_{n}^{\text{rest}}}$$

Here ${\text{EMG}}_{n}^{\text{rest}}$ and ${\text{EMG}}_{n}^{\text{max}}$ refer to the contraction levels of the muscle at rest and maximum contraction, respectively. The classification algorithm (LLGMN) formulated as

5 $$P\left(y|x\right)=\frac{\sum_{j=1}^{M}{\pi }_{j}{g}_{j}x}{\sum_{i=1}^{M}\sum_{j=1}^{M}{\pi }_{j}{g}_{j}x}$$

where $P\left(y|x\right)$ is the posterior probability of class $y$ given input $x, {g}_{j}(x)$, is the Gaussian mixture function, and ${\pi }_{j}$ is the weight associated with each mixture component.

**Fig. 5:**
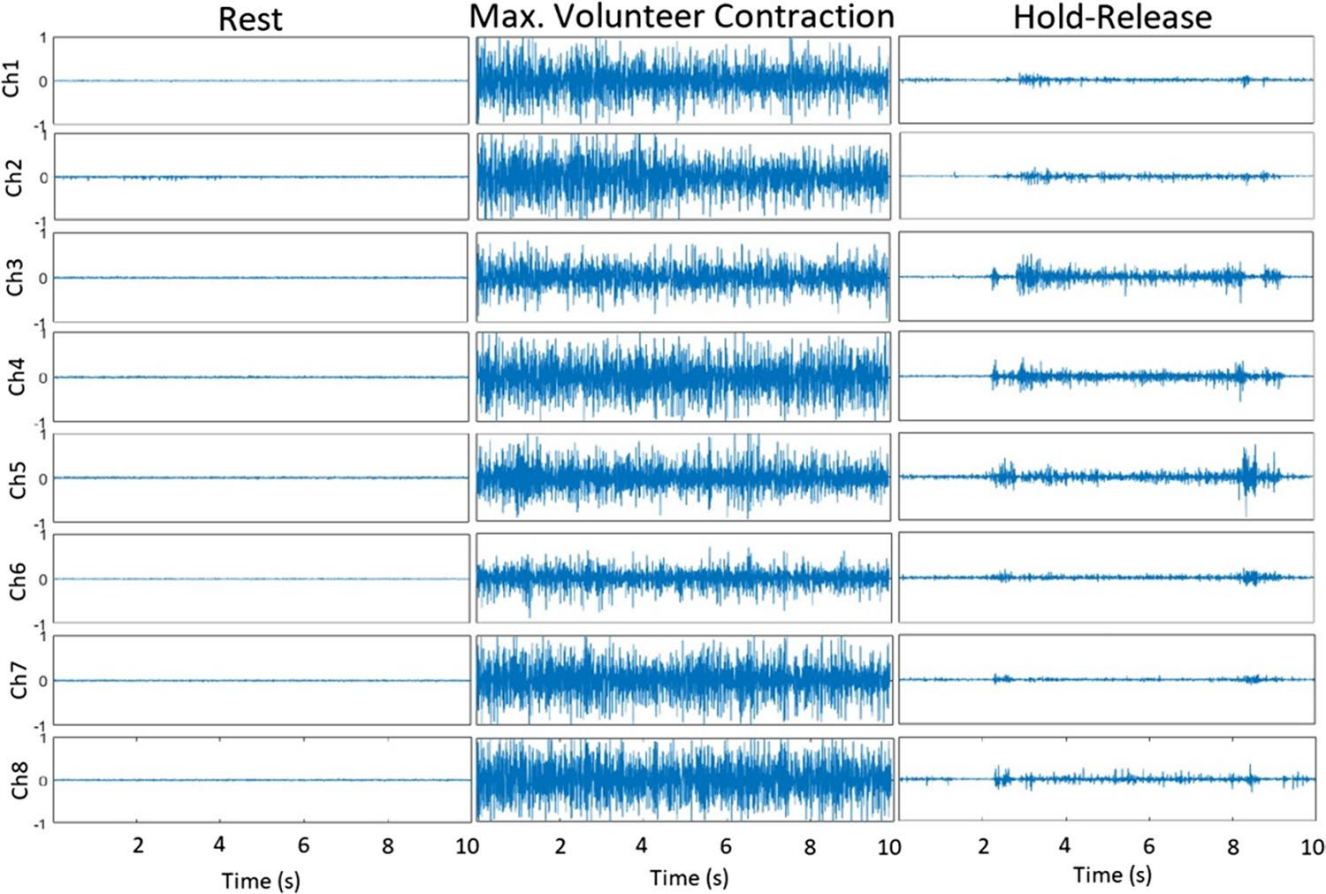
EMG signals during rest, maximum contraction, and hold-release

**Fig. 6:**
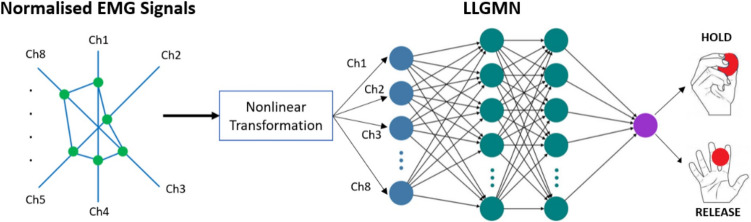
LLGMN network structure

The muscle contraction at rest was measured and recorded in a relaxed state, while the maximum muscle contraction was measured and recorded by squeezing the disc vigorously with the fingers. After signal preprocessing, normalization, and MCL calculation, data recording for each movement starts. The holding and releasing movements are performed between the second and sixth seconds of the 8-s recording period, and the EMG signals are recorded. An example signal is given in Fig. 5. From these recorded signals, 20 samples are selected and fed to the LLGMN model. The network is trained with this data and weights are determined. Classification is performed using these weights. With the motion information obtained at the output of the network, it is determined which of the holding and releasing movements are performed. The LLGMN network structure is given in Fig. 6.

### Analysis of arm movements

**Fig. 7:**
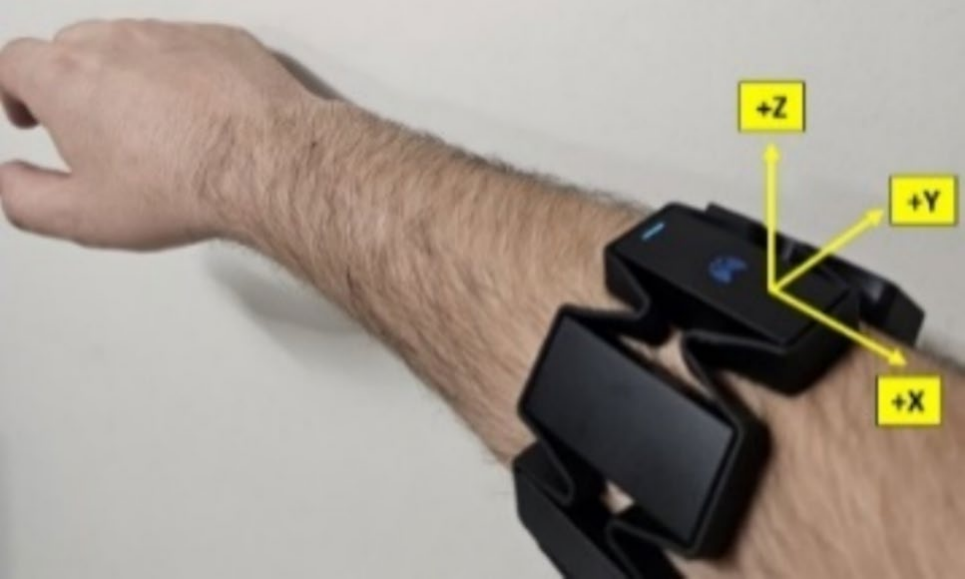
Axes on the MYO armband

In the developed system, in addition to movement classification, arm movements were also analyzed, and the movement speed and acceleration of the arm during the test were used in the evaluation phase. For example, if the arm is motionless or moving at low speeds while the participant does not have a disc in his/her hand, it can be concluded that he/she is free thinking. Arm speed and acceleration measurements were made with a 9-axis IMU on the MYO armband. The IMU consists of a 3-axis gyroscope (range: ± 2000°/s, nonlinearity: 0.2%, Total RMS noise: 0.06°/s), 3-axis magnetometer (range: ± 1200 µT), and three-axis accelerometer (range: ± 16 g, nonlinearity: 0.5%, total RMS noise: 4 mg). Angular velocities are given as roll, pitch, and yaw, which express rotation around the *X*, *Y*, and *Z* axes, respectively. Figure 7 shows the position of the MYO Armband device on the arm and the axis directions.

The acceleration is received at the sensor output in g and converted to m/s^2^. Acceleration values are recorded relative to a fixed set of axes. The gravitational acceleration of 9.81 m/s^2^ was subtracted from the acceleration measured in the *Z* direction. The average and maximum-minimum values of the angular velocity and linear acceleration data obtained from the IMU are presented to the experts via the interface.

### Creation of expert system rule base

In the system, disc hold and release times obtained as a result of motion classification with EMG, disc placing and turning times and total test time detected by the camera, angular velocity and acceleration values obtained from the IMU are evaluated in a rule base created with the information received from experts, and an inference is presented through the interface. The rule base was created with information from a five-person expert team consisting of two physical medicine and rehabilitation physicians and three physiotherapists. The expert system was defined using a set of inference rules. Each rule is represented as

6 $$R_i:IF\left(x_1\epsilon X_1\right)^\wedge\left(x_2\in X_2\right)^\wedge\dots^\wedge\left(x_n\in X_n\right)\;THEN(y\in Y)\;$$

Sample rules are given below.

**Rule** < *IF ****tt_p*** < ***max_t_p**** AND ****tt_y* > *min_t_p**** AND**** tt_t* < *max_t_t**** AND**** tt_t* > *min_t_t**** THEN Text* = *′Normal′* > 

For participants whose total test time in the two phases is between normal values, based on the experts’ recommendation, it will be sufficient to send a notification that the results are normal. Here, *tt_p* and *tt_t* represent the total test times in the placing and turning phases, *max_t_p*, *min_t_p*, *max_t_t*, and *min_t_t* represent the maximum and minimum normal limits in the placing and turning phases. These values are 62 s for the placing phase and 67 s for the turning phase. Normal limit values were determined according to the durations in the literature and the data obtained from 20 participants in this study. They can be changed when desired.

When the time in the placing and turning phases is above normal, the subcomponents of the test are checked by branching to the relevant functions and executing the rules there. For example, when the following rule is executed, the subcomponents of the placement phase are checked.

**Rule** < *IF ****tt_p*** < ***max_t_p**** AND**** tt_p* > *min_t_y**** AND**** tt_t* > *max_t_t**** AND**** tt_t* > *min_t_t*** > 

**Rule** < *IF ****rot_r_t* < *nrot_r_t**** THEN Text* = *′Insufficient rotation in turning. Possible restriction of pro-sup.′* > 

This sub-rule was activated when the participant completed the turning phase above normal time. As a subcomponent, the angular velocities of the arm are controlled. *rot_r_t* indicates the angular velocity in the roll direction during the turning phase, and *nrot_r_t* indicates the minimum level of the normal range of values for this velocity. If the angular velocity around the roll axis is below normal, it is indicated that the person has difficulty in the disc turning and that attention should be paid to the possibility of a limitation in the forearm pronation-supination movement.

**Rule** < *IF ****acc_x_p* < *nacc_x_p**** OR**** acc_y_p* < *nacc_y_p**** OR**** acc_z_p* < *nacc_z_p**** THEN Text* = *′Inadequate movement in placing. Possible restriction of the elbow and/or shoulder joint.′* > 

This sub-rule was activated when the participant completed the placing phase above the normal time. *acc_x_p*, *acc_y_p*, and *acc_z_p* indicate the average acceleration in the *x*, *y*, and *z* directions, respectively. If any of these accelerations are below normal, it is indicated that the person had difficulty in reaching and that attention should be paid to the possibility of a limitation in the elbow and/or shoulder joint.

**Rule** < *IF ****th_p* > *n_th_p**** THEN Text* = *′High inactive waiting time.'* > 

This sub-rule is activated when the participant remains stationary or moves at a low level during the placement. The angular velocity and accelerations from the IMU sensor are both recorded as thinking time (*th_p* or *th_t*). If this value is normally above the maximum level of the specified range, it should be understood that the person is stuck in the test for some reason. The possibilities of not understanding the test, having a stimulus in the environment that disturbs the participant, or having a cognitive problem should be evaluated.

**Rule** < *IF ****d16_t* > *n_d_t**** THEN Text* = *′Delay in turning on disc 16.′* > 

The sub-rules in the system check the placing and turning times of each disc. This sub-rule indicates that the participant takes longer than usual to turn the sixth disc. With this subset of rules, it can be displayed to the experts which discs are placed or turned in a longer time than normal.

In this section, example rules for different types of inferences are given and explained. More than one rule can be active for a participant at the same time. In this case, the inferences are listed on the user interface. For example, a participant may have both longer than normal disc turning times and longer than normal thinking times. The rule base of the system is open to improvement, and new rules can be added.

## Results and discussion

### Testing disc placing and turning times determination performance

**Fig. 8:**
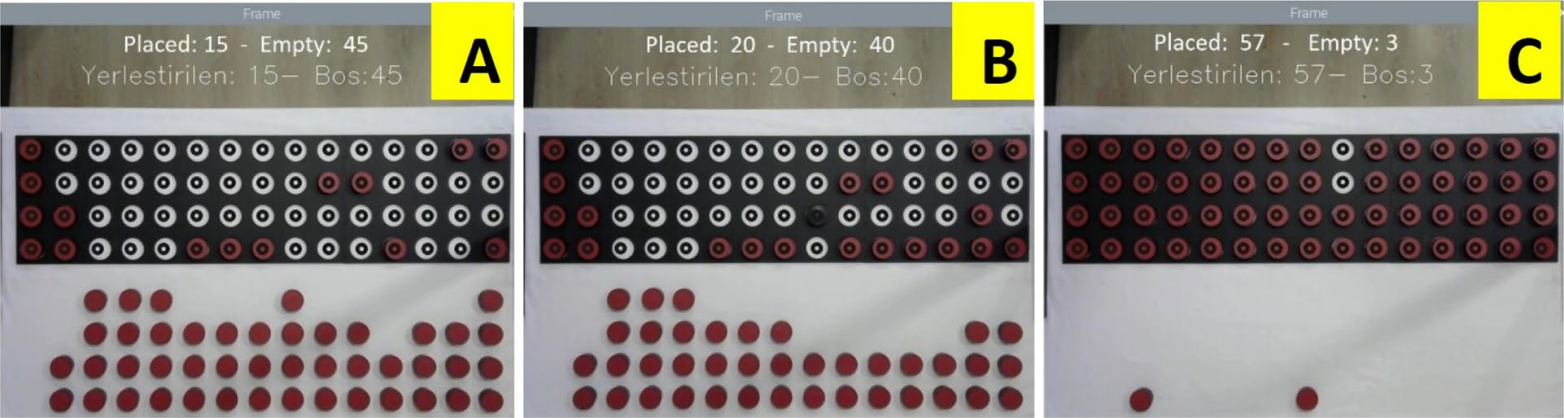
Trials for the disc placing phase

In the study, the placing and turning times of each disc were calculated by image processing. In this process, it is important to determine the disc orientation (whether the upward-facing face is red or black). For this purpose, 100 times of placing and 100 times of turning were tested under different lighting conditions. Figure 8 shows three of the combinations in the trials. The display shows a counter for the number of discs inserted and the number of empty holes. In Fig. 8a, 15 discs are placed in random locations. The counter again shows the correct values. In Fig. 8b, one disc was placed upside down with the black side up to determine whether the system made a mistake. The system again gave the correct result. In Fig. 8c, a shadow was created on the test board and the response of the system was analyzed. In this case, the system undercounted one disc. Since the color value in the center of the bottom right disc was closer to black, this disc could not be detected. When the shadow was removed, the correct result was obtained.

**Fig. 9:**
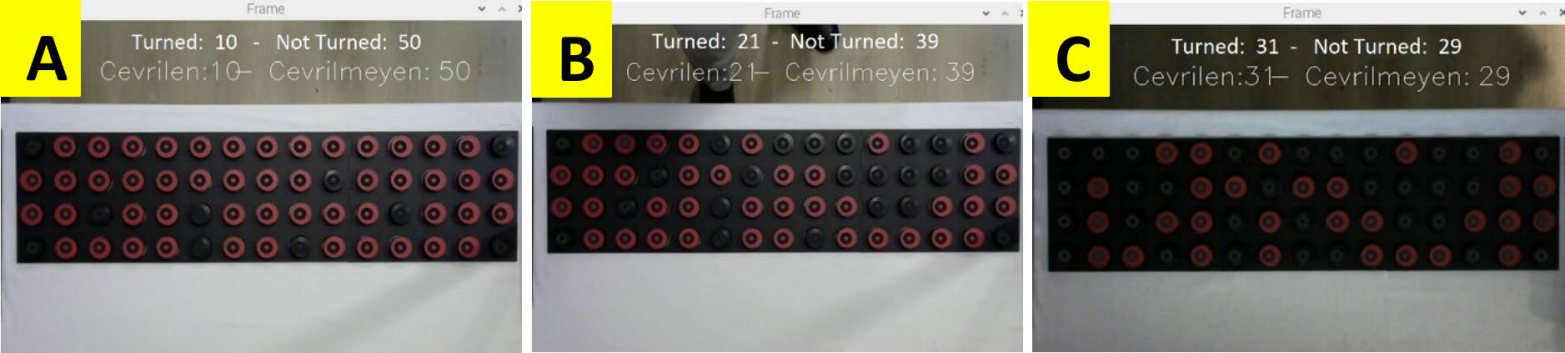
Trials for the disc turning phase

Figure 9 shows three of the combinations during the disc rotation phase. In Fig. 9a, 10 discs were randomly flipped. The counter again shows the correct values. In Fig. 9b, a flash was shone on the test board to simulate the ambient light change. As can be seen, flashes occurred on the side of the board facing the table edge. In this case, the system could not detect the black side of one of the discs and counted one disc as missing. When the flash was turned off, the correct result was obtained. In Fig. 9c, the ambient light level was reduced. In this case, no error occurred in the system and the correct number of discs was detected.

**Fig. 10:**
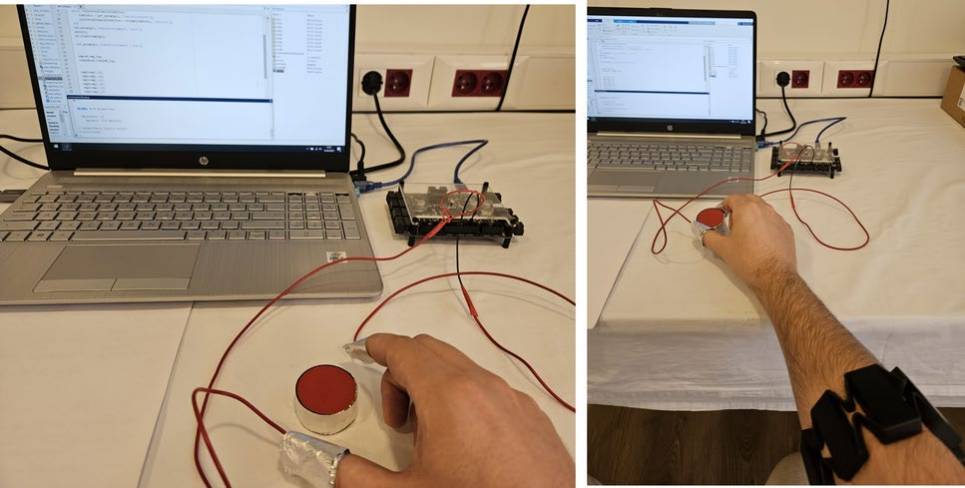
Motion classification performance test setup

In the 100 trials in the placing test, 89 correct and 11 incorrect results were obtained. In these trials, when the ambient light was normal, the correct results increased to 97 and 3 results remained incorrect. When the ambient light was changed in some of the 100 trials in the turning phase, 86 correct and 14 incorrect responses were obtained. In particular, when a strong light source was directed at the test board, reflection occurred on the black surface, resulting in incorrect results. On the other hand, reducing the ambient light did not have a negative effect. When the light was normal in these trials, the number of correct answers increased to 95. Five results remained incorrect. When the results are evaluated, a total of 87.5% accuracy was obtained in 200 trials in which the ambient light was manipulated. When the ambient light was left in its natural state, 96% accuracy was achieved in 200 trials.

### Testing the performance of hold-release motion classification

**Fig. 11:**
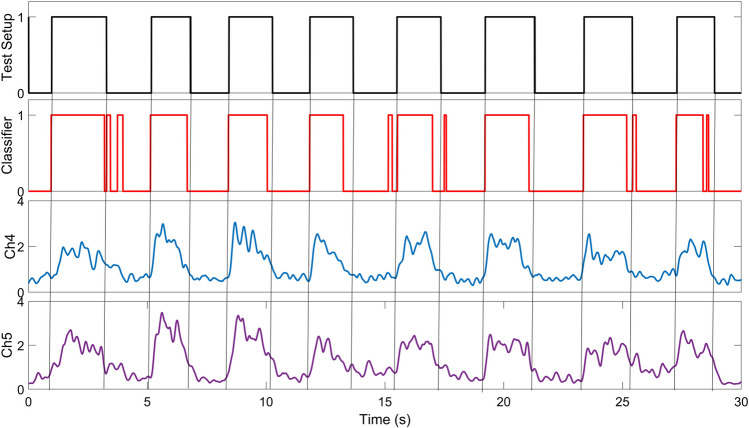
Comparison of test setup and classifier results

In order to test the motion classification performance of the system, the setup shown in Fig. 10 was prepared. Conductive aluminum foil was wrapped around the disc. Aluminum foil was also wrapped around the user’s thumb and index finger and the two ends of a wire were connected. When the gripping motion is performed, the wire ends are short-circuited through the conductive aluminum foil on the disc and the circuit is completed. Meanwhile, “1” information is transmitted to the computer via the digital input of the V-Daq real-time data acquisition card. In the release movement, the circuit is open and “0” information is transmitted. At the same time, EMG data is also recorded via the MYO armband. Motion classification is applied to the recorded EMG signals. The classification result is compared with the hold-release states obtained from the test setup. As an example, these 2 outputs in 8 hold and release movements performed for 30 s are compared in Fig. 11. At the same time, the muscle contraction levels seen in EMG channels 4 and 5, which are dominant in the hold and release motion, are also given. With the aforementioned procedure, 20 hold-and-release movements were performed in 25 sets, and a total of 500 hold-and-release movements were recorded. Each hold-and-release gesture was performed by the users at random durations. The results are given in Table 1.

**Table 1:** Comparison of disc hold times

Set	Total time (s)	Hold time (classifier) (s)	Hold time (test setup) (s)	Difference (%)
1	74	31.6	32.8	3.8
2	61	27.6	27.2	1.4
3	64	25.8	26.3	1.9
4	57	23.8	25.5	7.1
5	45	22.1	21.5	2.7
6	44	19.5	19.4	0.5
7	46	21.1	22	4.3
8	40	17.6	18.4	4.5
9	39	19	19.8	4.2
10	43	25.1	26.8	6.8
11	54	32.9	32.2	2.1
12	43	22.6	21.2	6.2
13	55	28.2	28.5	1.1
14	46	21.9	22.8	4.1
15	49	25.1	24.4	2.8
16	48	20.7	20.2	2.4
17	42	21.2	22.6	6.6
18	50	23.1	21.7	6.1
19	48	22.6	21.2	6.2
20	48	21.7	22.4	3.2
21	55	22.3	23.7	6.3
22	71	25.3	24.4	3.6
23	52	20.9	21.2	1.4
24	46	21.6	20.9	3.2
25	48	24.7	25.2	2.0

The total time column in Table 1 shows the time elapsed in each set (sum of hold-release), the hold time, HT (classifier), column shows the total hold time detected by the classification algorithm, and the hold time, HT (test rig), column shows the total hold time recorded by the test setup shown in Fig. 10. When the results are analyzed, it is seen that the classification algorithm reaches a minimum accuracy rate of 92.9% and a maximum accuracy rate of 99.5% when each set is analyzed separately. When these values are averaged, an accuracy of 96.2% is obtained.

The results obtained with the LLGMN algorithm are compared with the results obtained with feed forward neural networks (FNN) and support vector machine (SVM) methods. In [27], it was observed that mean absolute value (MAV) and integrated EMG (IEMG) features gave the highest accuracy value in non-real-time classification using SVM. Based on these results, MAV, IEMG, and root mean square (RMS) values were calculated from the recorded EMG data and the dataset was divided into training (15 sets) and test (10 sets). Experiments with FNN and SVM algorithms yielded the results given in Table 2. The eightfold cross-validation method was used to calculate the accuracies.

**Table 2:** Classification results for FNN and SVM algorithms

	FNN			SVM		
	RMS	MAV	IEMG	RMS	MAV	IEMG
Accuracy (%)	81.9 ± 2.5	88.6 ± 1.9	90.1 ± 1.7	88.7 ± 2.1	96.6 ± 1.5	96.3 ± 1.2

When the results are analyzed, it is seen that the SVM classifier has higher accuracy than the FNN. When compared with LLGMN, it is seen that both methods give similar results. High accuracy values could be obtained because the hold-and-release gesture by its nature produces more muscle contraction than other fine sensitive finger movements.

### Testing system performance with healthy participants

**Fig. 12:**
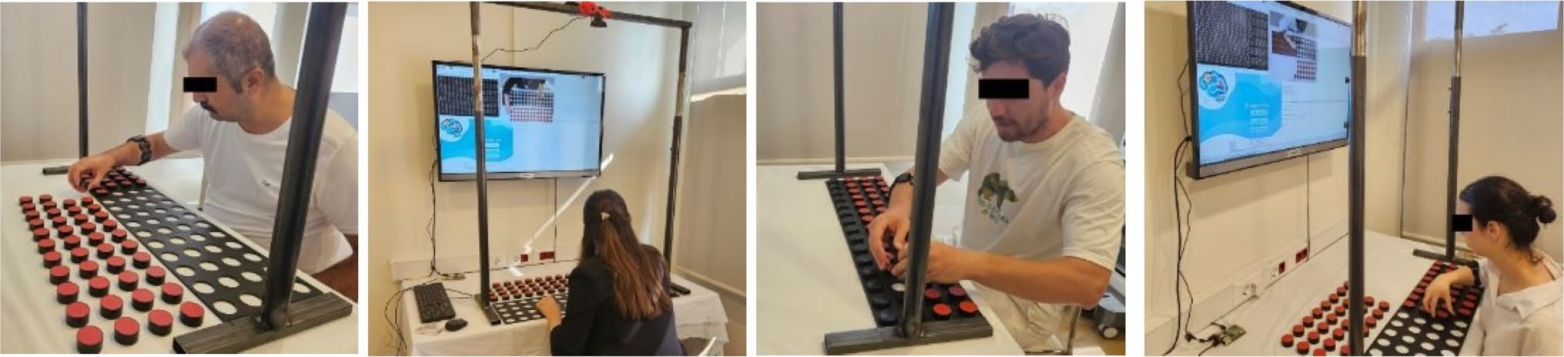
Images from the test of participants

In order to test the measurement performance of the developed system, trials were conducted with 20 healthy participants (10 women and 10 men). Fifteen of the participants were from the white-collar occupational group (engineers, academicians, and physicians), three participants were blue-collar (technicians and servants), and 2 participants were students. The inclusion criteria of the participants were 18–60 years of age, no history of upper limb nerve amputation, upper limb bone fracture, long-term (6 months or more) immobilization of the upper limbs, no muscle disease, and predominantly right-handed. In the test, 4 repetitions were made in each phase, one of which was a trial, and their average was taken in the calculation. The images taken during the test of healthy participants are shown in Fig. 12.

#### Analysis of disc placing and turning times

During the test, the placing and turning of the discs are monitored in real time by the camera, and the times are recorded for each phase. A second camera is used to record the tests. When the test is completed, the placing and turning times of each disc are calculated by timing the camera images with a stopwatch manually. The time error (${E}_{t}$) between system measurements and manual stopwatch measurements is calculated by Eq. 7.

7 $${E}_{T}=\frac{\left|{T}_{s}-{T}_{m}\right|}{{T}_{m}}100$$

Here, ${T}_{s}$ and ${T}_{m}$ are system-measured time and manually measured time, respectively.

Table 3 provides a detailed comparison of the average placing and turning times for each of the 60 discs, calculated from data obtained across 20 participants. For each disc, average ${T}_{s}$ and ${T}_{m}$ are presented. The time errors in the ${E}_{t}$ column in the table are the average of the disc placing and turning errors for each participant. The average placing times ranged from 0.79 to 1.29 s, while average turning times ranged from 0.89 to 1.46 s. Across most discs, the system and manual measurements showed strong agreement, with average time errors generally remaining below 10%. Notable deviations above this threshold, such as in disc 2 and disc 43 during the turning phase, may be attributed to transient visual obstructions or variations in hand motion that temporarily limited the camera’s field of view. Overall, the high temporal consistency between the system and manual measurements supports the accuracy and reliability of the developed system in tracking fine motor task timings, thereby demonstrating its potential utility for objective and automated dexterity assessment.

**Table 3:** Comparison of average disc placing, turning times, and average time error of 20 participants

	Placing			Turning				Placing			Turning		
Disc	${\overline{T} }_{s}$ *(s.)*	${\overline{T} }_{m}$ *(s.)*	${\overline{E} }_{T}$ *(%)*	${\overline{T} }_{s}$ *(s.)*	${\overline{T} }_{m}$ *(s.)*	${\overline{E} }_{T}$ *(%)*	Disc	${\overline{T} }_{s}$ *(s.)*	${\overline{T} }_{m}$ *(s.)*	${\overline{E} }_{T}$ *(%)*	${\overline{T} }_{s}$ *(s.)*	${\overline{T} }_{m}$ *(s.)*	${\overline{E} }_{T}$ *(%)*
1	1.15	1.13	11.02	1.44	1.38	5.30	31	1.12	1.12	4.08	1.17	1.21	5.51
2	0.99	0.97	5.02	1.21	1.08	14.04	32	1.13	1.14	4.97	1.50	1.49	5.80
3	1.07	1.09	3.83	0.96	0.93	8.34	33	0.94	0.93	7.65	1.36	1.36	5.76
4	1.24	1.25	5.18	0.90	0.90	4.14	34	0.91	0.87	6.15	1.14	1.12	7.21
5	1.02	1.00	4.78	1.02	1.00	7.64	35	1.02	1.04	5.86	1.03	1.04	6.21
6	0.93	0.91	6.86	1.13	1.12	7.30	36	1.25	1.22	4.05	0.97	0.94	6.26
7	1.08	1.09	4.03	1.10	1.08	5.35	37	0.79	0.78	4.91	1.01	0.98	5.60
8	1.25	1.25	3.91	1.25	1.24	4.04	38	0.85	0.84	4.45	0.98	0.95	6.74
9	0.99	0.99	4.06	1.07	1.07	8.05	39	1.06	1.08	6.07	0.93	0.96	6.84
10	0.94	0.91	5.49	1.27	1.25	6.36	40	1.14	1.13	2.91	0.96	0.98	6.33
11	1.00	1.01	5.52	1.24	1.22	8.28	41	0.97	0.99	6.69	0.96	0.95	10.00
12	1.14	1.17	5.27	1.20	1.20	5.21	42	0.92	0.91	5.69	0.98	0.98	6.51
13	0.93	0.91	4.80	1.19	1.20	6.15	43	0.94	0.94	7.66	1.10	1.05	10.50
14	0.96	0.97	5.66	1.18	1.18	5.49	44	1.08	1.05	5.82	0.98	1.00	4.82
15	1.09	1.10	4.82	1.28	1.25	4.64	45	0.94	0.91	5.78	1.24	1.26	4.94
16	1.22	1.21	5.19	1.46	1.46	5.37	46	0.87	0.87	4.54	1.40	1.41	5.97
17	0.89	0.89	5.80	1.21	1.24	5.54	47	0.92	0.92	5.15	1.29	1.29	4.88
18	0.87	0.84	5.88	1.12	1.11	6.83	48	1.09	1.09	3.60	1.02	1.00	9.19
19	1.09	1.09	3.76	1.19	1.17	7.14	49	0.88	0.85	5.17	1.08	1.12	5.40
20	1.28	1.27	4.16	1.05	1.10	5.90	50	0.96	0.96	5.24	1.03	1.06	4.60
21	0.89	0.88	5.70	1.04	1.04	4.40	51	0.93	0.93	5.16	0.97	1.02	6.27
22	0.92	0.90	5.34	1.09	1.04	5.45	52	1.03	1.01	6.16	1.05	1.05	3.77
23	1.16	1.15	4.99	1.03	1.05	6.50	53	1.05	1.03	6.54	0.97	0.98	5.22
24	1.29	1.24	5.51	0.99	0.98	6.20	54	0.98	0.97	5.85	1.04	1.02	3.79
25	0.92	0.89	7.21	1.14	1.12	5.35	55	1.07	1.06	4.32	1.01	0.99	5.18
26	0.87	0.88	5.41	1.03	1.02	5.74	56	1.02	1.05	5.69	1.08	1.05	6.77
27	1.07	1.06	4.83	1.15	1.12	5.55	57	0.96	0.96	4.39	1.09	1.11	5.08
28	1.13	1.13	4.49	1.11	1.13	7.67	58	1.02	1.02	6.68	0.89	0.90	6.72
29	0.94	0.96	7.62	1.11	1.14	4.44	59	1.08	1.08	4.89	1.03	1.08	5.24
30	0.86	0.83	6.67	1.19	1.16	5.35	60	1.09	1.09	6.70	1.15	1.19	8.99

#### Analysis of disc hold-release times

The time, muscle contraction levels, and the movement classification results obtained from them were recorded for the entire test. Hold-release times were obtained by subtracting the samples with values marked as hold as a result of motion classification from the samples obtained in the whole test. The results of the evaluation of EMG signals and motion classification for each participant during the placing and turning are given in Table 4. The times were determined by averaging 3 repetitions in each phase.

**Table 4:** Comparison of disc hold times in placing and turning phases

	Placing			Turning		
No	Total time (s)	Hold time (s)	**%**	Total time (s)	Hold time (s)	**%**
P1	62	28.3	45.6	66	30.9	46.8
P2	65	27.1	41.7	69	29.7	43.0
P3	63	34.7	55.1	67	32.1	47.9
P4	57	25.4	44.6	66	28.4	43.0
P5	61	29.8	48.9	76	29.3	38.6
P6	55	19.7	35.8	58	27.3	47.1
P7	56	26.5	47.3	62	33.5	54.0
P8	62	26.1	42.1	67	29.9	44.6
P9	60	32	53.3	64	27.1	42.3
P10	58	31.7	54.7	67	24.3	36.3
P11	59	27.9	47.3	62	30.1	48.5
P12	59	25.1	42.5	67	25.7	38.4
P13	67	24.8	37.0	77	26.1	33.9
P14	64	24.9	38.9	69	35.2	51.0
P15	63	19.6	31.1	69	29.3	42.5
P16	63	24.4	38.7	70	30.3	43.3
P17	68	36.9	54.3	71	26.7	37.6
P18	60	27.3	45.5	64	28.7	44.8
P19	66	23.8	36.1	67	23.1	34.5
P20	65	33	50.8	71	34.8	49.0
**Mean**	**61.7**	**27.5**	**44.6**	**67.5**	**29.1**	**43.4**
**Max**	**68**	**36.9**	**55.1**	**77**	**35.2**	**54**
**Min**	**55**	**19.6**	**31.1**	**58**	**23.1**	**33.9**
**Std. dev**	**3.6**	**4.5**	**7**	**4.5**	**3.2**	**5.5**

When Table 4 is examined, it is seen that the average time taken by the participant while the disc was in his/her hand during the placing phase conducted with 20 participants was 27.5 s. This result corresponds to 44.6% of the total average time. Considering the nature of the test, in the placing, the participant reaches out his/her hand, grabs the disc, and places it in its place, and the disc remains in his/her hand for almost half of the total time. Therefore, the result is compatible with the structure of the test. In the turning phase, the participant picks up the disc with one hand, turns it, and places it in place by passing it to the other hand. In each row of the test board, he changed the hand with which he grasped and turned the disc. When the test results were analyzed, it was found that the average time taken during the turning phase was 29.1 s. This result corresponds to 43.4% of the total average turning time. When the nature of the test is examined, the discs were grasped for most of the process. However, the average of the times obtained was below 50%. This time was lower than expected because only the dominant hand to which the MYO armband device was connected was measured. It is thought that the time will increase to the expected value when the EMG device is connected to both arms.

#### Analysis of arm movements

Wearable sensors for non-invasive detection of limb movements that occur during various activities are widely used today. These sensors overcome many of the drawbacks of camera-based systems, which are considered the gold standard for motion analysis, allowing the application of motion in its natural environment [28–30]. Inertial sensors [30, 31] are widely used to objectively measure body kinematics during various activities in a way that does not restrict movement. In this study, arm movements during dexterity tests were measured with the IMU sensor to detect possible movement limitations of individuals during the test and to suggest the diseases that should be focused on accordingly to the experts. With gyroscope data, roll, pitch, and yaw values were obtained. Table 5 shows the maximum angular velocity values for the placing and turning phases of the test.

**Table 5:** Angular velocities of the arm during the disc placing and turning phase

	Roll (°/s)		Pitch (°/s)		Yaw (°/s)	
No	Placing	Turning	Placing	Turning	Placing	Turning
P1	94.5	117.5	104	91	193	40.5
P2	153	132	133.5	86.5	222.5	68
P3	70.5	90	132.5	72.5	183	45
P4	135.5	102.5	170.5	68.5	177	96
P5	140.5	130	114.5	58	159.5	84.5
P6	176.5	164	261.5	75.5	177.5	155
P7	119	89	166.5	106	239.5	51
P8	123	130	113.5	118	211.5	52.5
P9	111	81.5	88	80	172	35.5
P10	131	120.5	161	87	199	59
P11	136.5	96.5	164	99	229	48
P12	124.5	126	153	93.5	191	50.5
P13	166	120	106.5	59.5	171.5	47.5
P14	148.5	115	159.5	95.5	203	46
P15	131	91.5	118	115.5	163.5	81
P16	136.5	92.5	123	116	200.5	81
P17	109.5	106.5	167	79.5	175.5	60
P18	130	120.5	103.5	91	170.5	62.5
P19	110.5	92.5	152	66.5	171.5	47
P20	125	83	122	93.5	223	54.5
**Mean**	**128.6**	**110**	**140.7**	**87.6**	**191.6**	**63.3**
**Max**	**176.5**	**164**	**261.5**	**118**	**239.5**	**155**
**Min**	**70.5**	**81.5**	**88**	**58**	**159.5**	**35.5**
**Std. dev**	**23.7**	**21**	**38.3**	**17.9**	**23.5**	**26.9**

When the data in Table 5 are examined, the average angular velocities in the positive and negative directions in the roll, pitch, and yaw axes for the placing phase were 125, 122, and 93.5, respectively, while these values were 83, 93.5, and 54.5 in the turning phase. When the angular velocity results for arm movements are analyzed, it is seen that the arm moves at smaller speeds in the turning phase. This is an expected result since the discs are rotated in the position they are in during the turning, the fingers are mostly moving, and no reaching movement occurs.

Table 6 shows the linear accelerations during the placing and turning phases. Average accelerations were determined by averaging the peak values for each direction. When the data in the table are examined, the average accelerations in the *X*, *Y*, and *Z* axes for the placing were 2.1, 2.7, and 1.3, respectively, while these values were 0.9, 1.2, and 0.8 in the turning phase. When the linear accelerations of the arm in the placing and turning were examined in general, it was observed that the values were close to each other and the standard deviation was low. This means that these values can be used as a reference for the movements of healthy individuals during the dexterity test and inferences can be made on the test results of the patients. The motion analysis results of healthy individuals are suitable for the reference values in the expert system rule base. In the future, more accurate reference values can be obtained by increasing the number of healthy participants from 20 and expanding the database.

**Table 6:** Average linear accelerations of the arm during the disc placing and turning phases

	***X*** axis (m/s^2^)		***Y*** axis (m/s^2^)		***Z*** axis (m/s^2^)	
No	Placing	Turning	Placing	Turning	Placing	Turning
P1	2.35	0.8	2.5	1.25	1.05	0.75
P2	2.05	1.1	3.1	0.9	1.4	1.05
P3	2.25	1.15	1.75	0.7	1.1	0.8
P4	2.4	1.15	3.4	0.9	1.2	0.65
P5	2.95	0.75	1.4	0.9	0.95	0.65
P6	2.5	1.5	4.45	1.95	1.75	1.05
P7	1.65	0.9	2.5	1.05	1.3	0.75
P8	1.3	0.75	2.5	0.8	1.2	0.8
P9	1.35	0.95	3.3	1.4	1	0.9
P10	2	1.25	2.9	1.45	1.95	1.3
P11	2.45	0.8	1.85	1.2	1.1	0.85
P12	1.75	0.65	3.45	1.05	1.35	0.65
P13	1.75	0.7	2.7	0.9	1.1	0.6
P14	1.65	0.85	1.95	0.65	1.2	0.65
P15	2.7	1	3.35	1.25	1.35	0.7
P16	2.25	0.75	2.35	1.55	1.4	0.75
P17	2	0.95	2.95	1.35	1.15	0.8
P18	1.9	0.65	2.35	1.8	1.5	0.95
P19	1.95	0.7	1.9	0.95	1.45	0.7
P20	1.85	1.1	2.7	1	1.2	0.9
**Mean**	**2.1**	**0.9**	**2.7**	**1.2**	**1.3**	**0.8**
**Max**	**2.9**	**1.5**	**4.5**	**1.9**	**1.9**	**1.3**
**Min**	**1.3**	**0.7**	**1.4**	**0.6**	**0.9**	**0.6**
**Std. dev**	**0.43**	**0.23**	**0.73**	**0.35**	**0.25**	**0.17**

## Conclusions

In this study, a system that can automatically perform hand dexterity test with a multi-sensor structure consisting of EMG, IMU, camera, and artificial neural network-based motion classification algorithm has been developed. In the system, the muscle contraction levels occurring during the hand dexterity test are taken from the MYO armband placed on the forearm and evaluated with the artificial neural network-based LLGMN algorithm, and the hold-release motion is classified. Angular velocity and linear acceleration values obtained from the 9-axis IMU sensor on the same device were used for arm motion analysis. Disc movements were tracked with a camera placed on the test table, and the placing and turning times of each disc were determined. The data was evaluated using a rule base created with the information received from experts, and inferences were made about the condition of the individuals. Thanks to this system, it will be easier to meet the demands, especially in populated areas. Human measurement errors will be minimized. By measuring durations and movement dynamics that cannot be measured by humans, it will ensure that the subcomponents of skill tests can also be used in diagnosis and treatment stages.

There are several limitations in the study. Shadows on the test board, lack of ambient light, and light direction negatively affect the image processing performance. To solve this problem, various changes are planned to be made in the position of the camera and the table on which the test board is placed. In the system, images are processed in Python program on Raspberry Pi, and EMG and motion data are processed in MATLAB program on the computer. By organizing all these data processing stages to run on a single controller, a compact embedded system will be obtained with less hardware. Another limitation of the system is that it is not possible to detect whether users have dropped discs. For this, it is planned to provide a solution based on image processing or with a sensor to be placed on the discs.

## Data Availability

The data that supports the findings of this study are available from the corresponding author, upon request.
